# Evaluation of hair surface structure and morphology of patients with lichen planopilaris (LPP) by atomic force microscopy (AFM)

**DOI:** 10.1111/srt.70030

**Published:** 2024-09-01

**Authors:** Karolina Krawczyk‐Wołoszyn, Magdalena Żychowska, Adam Reich

**Affiliations:** ^1^ Doctoral School University of Rzeszow Rzeszów Poland; ^2^ Department of Dermatology Institute of Medical Sciences Medical College of Rzeszow University Rzeszów Poland

**Keywords:** AFM, atomic force microscope, atomic force microscopy, dermatology, FFA, frontal fibrosing alopecia, human hair, lichen planopilaris, LPP, trichoscopy

## Abstract

**Background:**

Lichen planopilaris (LPP) is a chronic lymphocytic skin disease manifested by progressive scarring alopecia. The diagnosis of LPP is made based on histopathological examination, although it is not always definite. The current study evaluates the effectiveness of non‐invasive atomic force microscopy (AFM) hair examination in detecting morphological differences between healthy and diseased hair.

**Materials and methods:**

Here, three to five hairs from lesional skin of 10 LPP patients were collected and examined at nine locations using AFM. At least four images were taken at each of the nine sites. Metric measurements were taken and metric (length, width, and scale step height) and morphological features (striated and smooth surface of scales, the presence of endocuticle and cortex, shape of scales edges, scratches, pitting, cracks, globules, and wavy edge) were compared with hair from healthy controls. In addition, areas on diseased hair where the process of pathological, unnatural delamination of the hair fiber occurs are described.

**Results:**

There was a statistically significant difference in the number of scratches in the initial sections of the LPP hair, in the intensity of wavy edges along the entire length of the tested hair, and in the number of scales with pitting in the middle section of the hair. In addition, a statistically significant higher number of scales with striated surface was found in LPP group starting at 3.5 cm from the root continuing towards the free end of the hair. Other morphological changes such as presence of cortex, globules, oval indentations, and rod‐like macrofibrillar elements were also assessed, however, detailed results are not presented, as the differences shown in the number of these morphological changes were not significantly different.

**Conclusion:**

This publication outlines the differences between virgin, healthy Caucasian hair, and the hair of LPP patients. The results of this study can be used for further research and work related to LPP. This is the first attempt to characterize the hair of LPP patients using AFM.

## 1 INTRODUCTION

Lichen planopilaris (LPP) is a primary lymphocytic skin disease with a multifactorial, incompletely understood pathogenesis. An autoimmune basis has been postulated. An inflammatory infiltrate of the scalp skin leads to damage of the hair follicle structures leading to atrophy and fibrosis. A band‐like subepidermal perifollicular lymphocytic infiltrate located in the follicular isthmus and infundibulum is seen in histopathology, although the histopathology is not always diagnostic. The disease is manifested by progressive patches of scarring alopecia. Clinically, there is an active inflammation at their periphery, which presents as perifollicular erythema and scaling. An additional symptom includes hyperkeratosis of the hair follicle, which causes exfoliation, creating a kind of ‘collar’ on the proximal part of the hair. Currently, the diagnosis of LPP is made based on histopathological examination from an invasive scalp biopsy and, supportively, on widely available and practical dermoscopy, although these examinations do not necessarily have to be conclusive.^1^, ^2^ Currently, the use of novel non‐invasive procedures such as optical coherence tomography (OCT) and reflectance confocal microscopy are being studied as an aid in the diagnosis.^3^, ^4^ In recent years, research has also begun on healthy human hair using atomic force microscopy (AFM).^5^, ^6^, ^7^ The employment of the AFM technique for nanoscale hair examination may contribute to improved diagnosis and treatment of scalp diseases.^8^ To date, a limited studies have been conducted on AFM imaging of hair diseases, limited to psoriasis, alopecia areata, and seborrheic dermatitis.^9^, ^10^, ^11^ The current study evaluates the potential usefulness of AFM hair examination in detecting morphological differences between healthy and LPP hairs. It has been hypothesized that due to the lymphocytic folliculitis and hyperkeratinization of the follicle's orifices, the hair morphology might differ from normal hair.

## 2 MATERIAL AND METHODS

The study used the method established in the authors' previous study on healthy, virgin Caucasian hair.^12^

### 2.1 Subjects

From a group of forty patients with a histopathologically confirmed diagnosis of LPP, 10 patients with the highest disease severity were selected (Table 1). The disease severity was assessed by physical examination, dermoscopy, and subjective symptoms. Then, three to five hairs, from the periphery of active lesions, were collected from each subject. The control group consisted of 10 healthy Caucasian subjects who had not dyed their hair for at least 3 months prior to the study (Table 1). In this case, three to five hairs were taken from the same skull areas as in LPP patients.

**TABLE 1:** Comparison of patients suffering from lichen planopilaris and healthy controls.

Characteristics	Study group	Control group	*p*
Sex, *n* (%)			
Women	9 (90.0)	9 (90.0)	N/A
Men	1 (10.0)	1 (10.0)	
Age (years), mean ± standard deviation (SD)	56.1 ± 10.0	43.8 ± 15.5	0.11
Range (years)	39–71	26–72	
Race, *n* (%)			
Caucasian	10 (100.0)	10 (100.0)	N/A
Age of diagnosis (years) mean ± SD	51.4 ± 14.7	**–**	N/A
Range (years)	15–66		
Disease duration (months) mean ± SD	56.0 ± 103.1	**–**	N/A
Range, months	4–360		
Clinical subtype of LPP, *n* (%)			
Classic LPP	5 (50.0)	**–**	N/A
Classic FFA	3 (30.0)		
Coexistence of LPP and FFA	2 (20.0)		
Locations outside the scalp, *n* (%)			
Involvement of the mucous membranes	0 (0.0)	**–**	N/A
Involvement of the nails	1 (10.0)		
Involvement of the face and armpits	1 (10.0)		

Abbreviation: N/A, not applicable.

### 2.2 Hair preparation

The clean hairs were pulled out along with their roots and subsequently cut vertically with a scalpel into 3 cm long sections. They were then attached to a microscope slide using a translucent tape.

### 2.3 Atomic force microscopy (AFM)

AFM was performed using a DriveAFM microscope with CX controller (Nanosurf, Liestal, Switzerland) under atmospheric conditions (in air) at room temperature and stable humidity (65%–70%). Imaging was performed in contact mode using PPP‐FMAuD‐10 tips (NANOSENSORS, Neuchatel, Switzerland) mounted on cantilevers with a constant force of 1.9 N/m and frequency of 75 kHz. Each hair was imaged at 0.5, 1.0, 1.5, 2.0, 3.5, 4.5, 5.5, 6.5, and 7.0 cm from the root. The scanning range covered an area from 40×40 µm to 3×3 µm. The scanning velocity was 0.5 s/line. The resolution of the images was 512−1024 points/line.

The images were computer‐processed using Nanosurf CX ver 3.10.3.7. The AFM tip was centered to be on top of the hair fiber upon contact with the cuticle surface. The AFM tip scanned the hair perpendicular to the longitudinal axis of the fiber to reduce errors due to the AFM tip hitting the sides of the hair. For each of nine locations (0.5, 1.0, 1.5, 2.0, 3.5, 4.5, 5.5, 6.5, and 7.0 cm from the root), at least four images were taken. Line profile creation, 3D visualizations, image processing, and all metric measurements (length, width, and scale step height) were performed using Gwyddion (64 bit) software ver 2.63. Line profiles were created by averaging data from 10 adjacent scan lines.

Assessment of morphological lesions on the surface of scales was performed manually on images of the same size to compare features between patients. The number of scales with striated surface and the scratches on the surface of scales were counted. Then, the above parameters were grouped on a scale from 0 to 5 as shown in Table 2. Parameters such as endocuticle, broken edges of scales shape of edges of scales, wavy edges of scales, the number of scales with smooth surface, presence of cortex, pitting, globules, oval indentations, and rod‐like macrofibrillar elements were evaluated in a descriptive manner by grouping a given characteristic in a range, as shown in Table 2.

**TABLE 2:** Quantitative and descriptive representation of crucial features in the LPP patient group.

Feature	Absolute number‐range or qualitative description of the feature	Corresponding grade
The number of scales with striated surface	0	0
	1–3	1
	4–7	2
	8–11	3
	12–15	4
	>15	5
Scratches	0	0
	1–9	1
	10–19	2
	20–29	3
	30–39	4
	>40	5
Endocuticle	Absent	0
	Single nodules 2–4 nm in diameter, located at the base of the scales’ edges.	1
	Single nodules spread over the entire scale surface.	2
	Granular plaques 3–5 nm wide localized at the base of the scales’ edges.	3
	Granular plaques 4−7 nm wide located across the width of the scales, at the base of the scale edges.	4
	Granular structure occupying more than 50% of the cuticle surface.	5
Broken edges	Edge undamaged, smooth.	0
	Edge smooth with single small cracks.	1
	Numerous small cracks, smooth edge visible for less than 40%.	2
	Lack of smooth edge, numerous larger cracks, and scale breakages.	3
	Deep breakouts, edge like chain saw's teeth.	4
	Very uneven, deep, different shaped breakouts.	5
Shape of edges	Convex	1
	Streight	2
	Concave	3
Wavy edges	Edge undamaged, smooth.	0
	Gentle undulation, U‐shaped wave ridges.	1
	Deeper and more marked undulations.	2
	Increased undulations and breaks between individual wave crests.	3

### 2.4 Statistical analysis

Statistical analysis was performed using Statistica software v.13.0 (TIBCO Software Inc., Kraków, Poland). Mean, minimal, and maximal values along with the standard deviations were calculated for longitudinal parameters. Student *t* test, Mann‐Whitney *U* test, or Chi^2^ test was used as appropriate. *p* values less than 0.05 were considered significant.

## 3 RESULTS

### 3.1 Dimensions and size of the hair scales

In the study group, the scale step height increased with distance from the root. The values of the apparent scale length and the scale width also increased. The tendency for these dimensions to increase was more noticeable in the study group than in the control group. However, the differences between the scale dimensions of the study group and the control group were not statistically significant. The measurements obtained are shown in Table 3.

**TABLE 3:** Metric measurements of healthy and diseased hair scales.

Distance from the root	Scale step height (µm)		*p*	Apparent scale length (µm)		*p*	Scale width (µm)		*p*
	LPP	Controls		LPP	Controls		LPP	Controls	
0.5 cm	0.5 ± 0.18	0.51 ± 0.1	0.83	7.1 ± 1.9	7.9 ± 1.1	0.26	19.3 ± 6.8	17.2 ± 3.6	0.4
1.0 cm	0.5 ± 0.16	0.51 ± 0.12	0.88	7.0 ± 2.0	6.8 ± 1.4	0.87	19.5 ± 5.6	16.6 ± 2.2	0.16
1.5 cm	0.51 ± 0.16	0.48 ± 0.11	0.64	7.0 ± 1.8	7.6 ± 1.4	0.45	19.6 ± 4.8	17.2 ± 2.6	0.18
2.0 cm	0.5 ± 0.17	0.49 ± 0.08	0.88	6.5 ± 1.2	7.7 ± 1.1	0.03	22.3 ± 5.1	20.6 ± 3.7	0.42
3.5 cm	0.61 ± 0.14	0.52 ± 0.05	0.11	6.7 ± 0.8	6.7 ± 1.0	0.96	21.8 ± 5.6	19.2 ± 2.9	0.21
4.5 cm	0.59 ± 0.16	0.53 ± 0.17	0.44	7.5 ± 1.4	7.9 ± 1.9	0.65	22.2 ± 5.3	19.2 ± 4.0	0.18
5.5 cm	0.67 ± 0.2	0.53 ± 0.09	0.06	7.7 ± 1.8	7.5 ± 1.3	0.79	21.9 ± 5.2	18.7 ± 3.2	0.12
6.5 cm	0.61 ± 0.14	0.53 ± 0.11	0.16	7.7 ± 1.8	7.3 ± 1.3	0.58	21.2 ± 6.1	20.2 ± 4.4	0.68
7.0 cm	0.63 ± 1.62	0.5 ± 0.13	0.07	7.8 ± 2.1	7.6 ± 1.6	0.83	21.2 ± 5.7	19.7 ± 4.3	0.52

Results demonstrated as means and standard deviations.

Abbreviation: LPP, Lichen planopilaris.

### 3.2 Comparison of morphological features between the study and control groups

**FIGURE 1:**
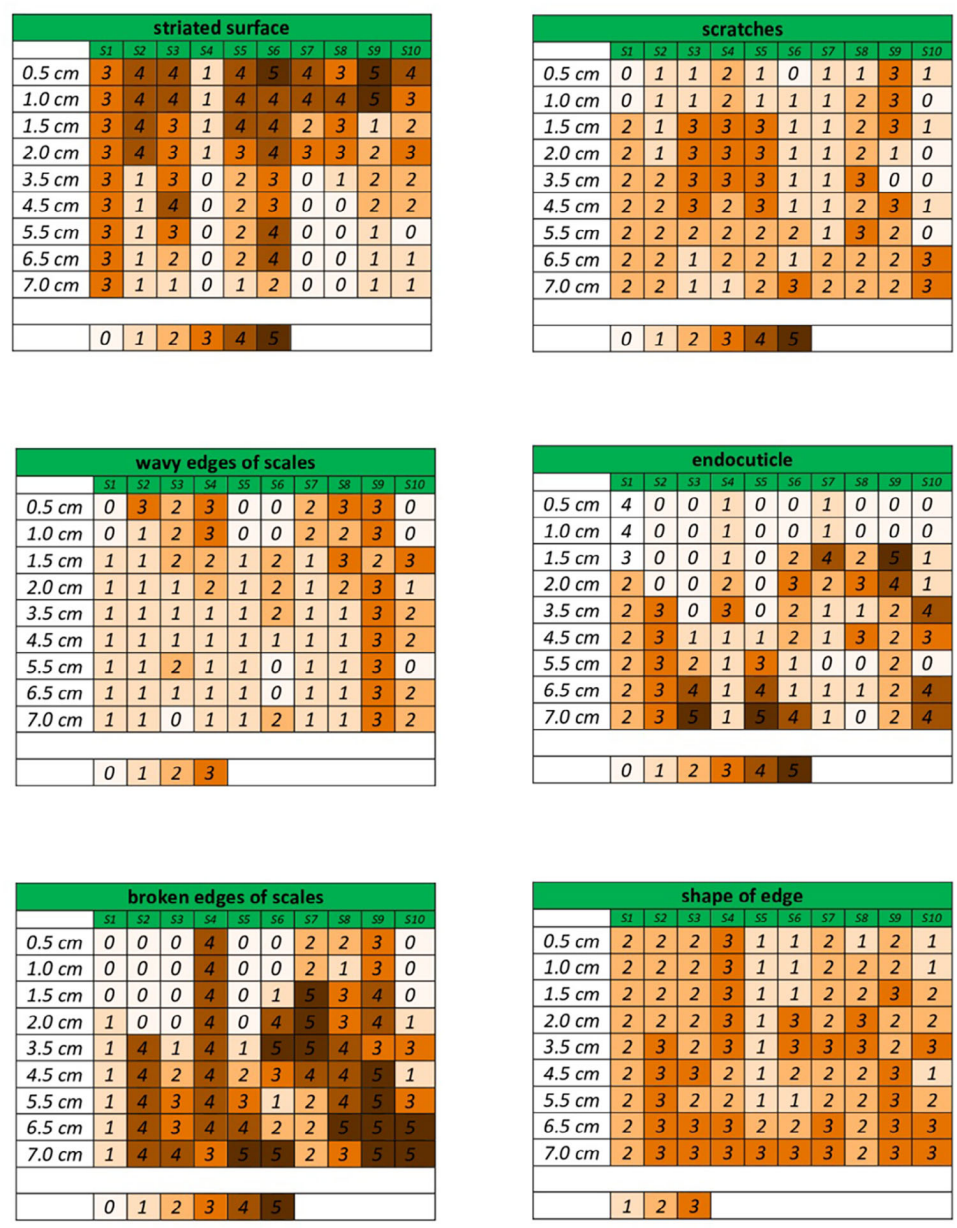
Distribution of crucial features of hairs obtained from patients with lichen planopilaris (scoring of parameters described in Table 2).

Features mentioned in Table 2 were analyzed, as already done in our previous publication on the evaluation of virgin Caucasian hair.^12^ Figure 1 shows the distribution of the most notable features in the hair surface morphology of LPP patients. The scoring of the features is characterized in Table 2.

**FIGURE 2:**
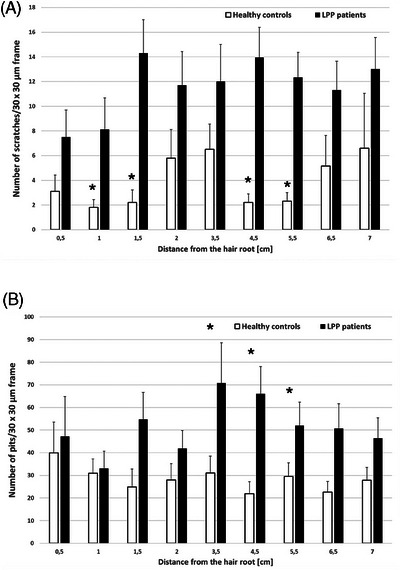
Comparison of lichen planopilaris hairs and healthy hairs regarding the (A) number of scratches, (B) number of pits within the hair scales (results demonstrate as means and standard errors, ^*^ *p* < 0.05).

**FIGURE 3:**
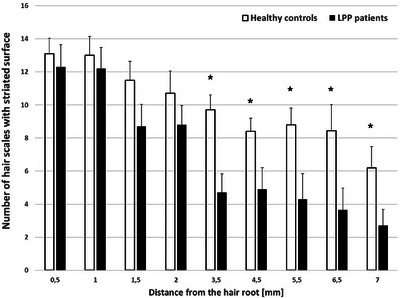
Comparison of lichen planopilaris hairs and healthy hairs regarding the number of scales with striated surface (results demonstrate as means and standard errors, ^*^ *p* < 0.05).

There was a statistically significant difference in the number of scratches in the initial sections of the LPP hair (1–5.5 cm) (Figure 2A), in the intensity of wavy edges along the entire length of the tested hair (0.5–7 cm), and in the number of scales with pitting in the middle section of the hair (3.5–6.5 cm) (Figure 2B). In addition, a statistically significant higher number of scales with striated surface was found in LPP group starting at 3.5 cm from the root continuing toward the free end of the hair (Figure 3). Other morphological changes such as presence of cortex, globules, oval indentations, and rod‐like macrofibrillar elements were also assessed; however, detailed results are not presented, as the differences shown in the number of these morphological changes were not statistically different.

**FIGURE 4:**
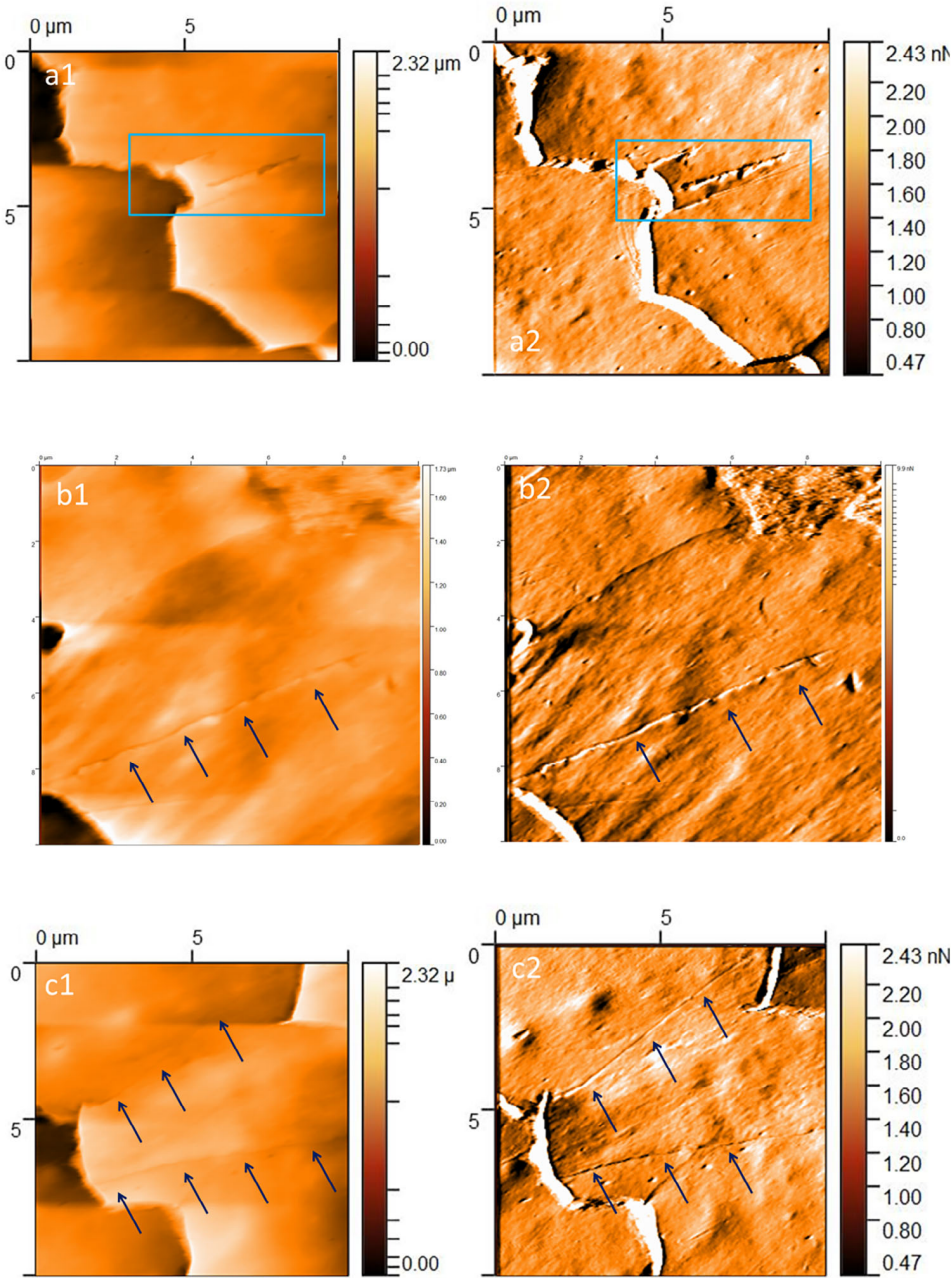
Atomic force microscopy of the hair from patient with lichen planopilaris. (a1‐a2) 3.5 cm from the root end. Deep linear scratches (blue frames). Image size 10 × 10 µm; a1 *Z*‐axis image; a2 deflection image. (b1‐b2) 2 cm from the root end. Long linear scratches (black arrows). Images size 10 × 10 µm; b1 *Z*‐axis image; b2 deflection image. (c1‐c2) 2 cm from the root end. Long linear scratches (black arrows). Images size 10 × 10 µm; c1 *Z*‐axis image; c2 deflection image.

**FIGURE 5:**
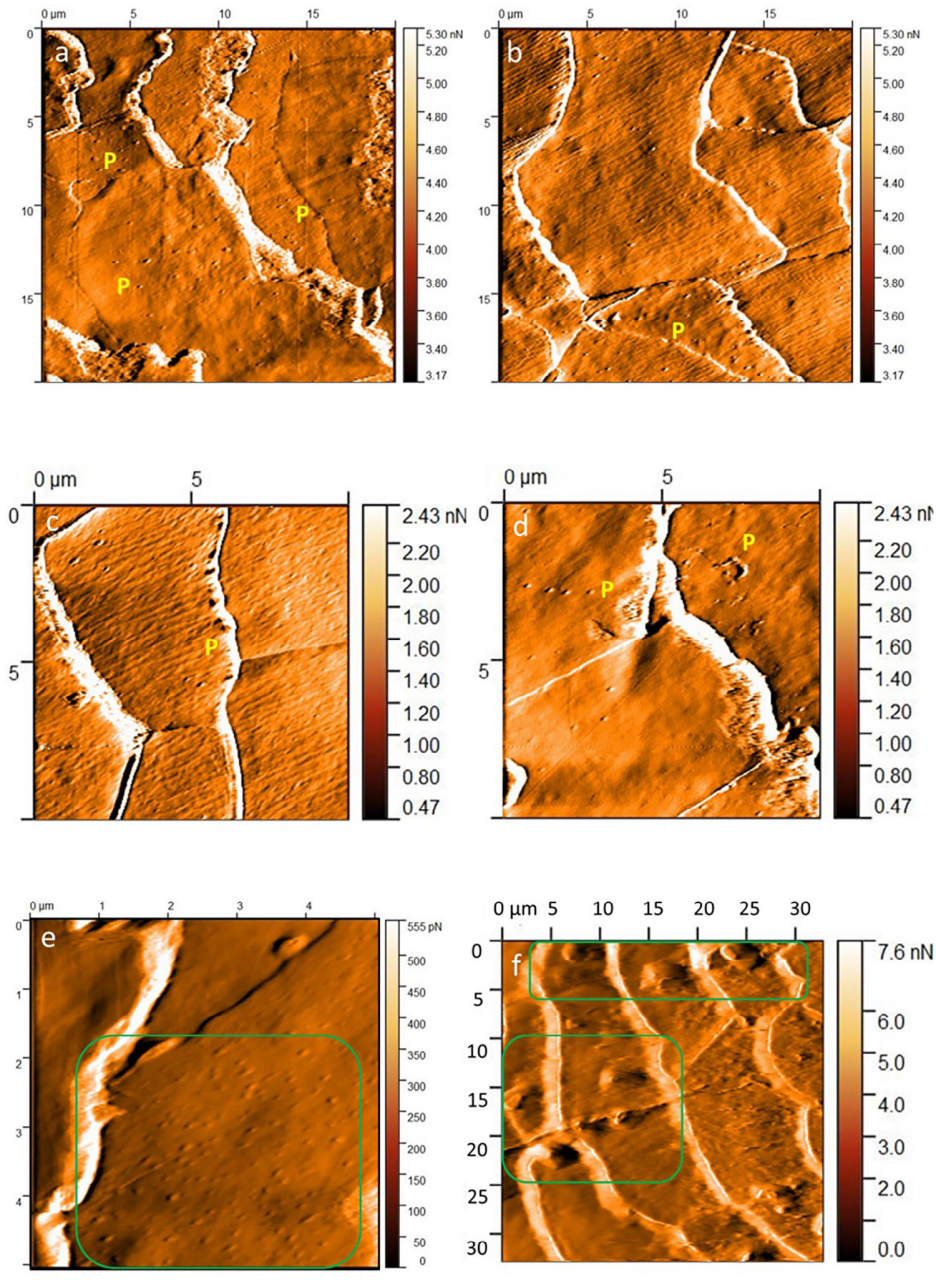
Atomic force microscopy of the hair from patient with lichen planopilaris. (A) 4.5 cm from the root end. Pitting (P). Image size 20 × 20 µm; deflection image. (B) 2.0 cm from the root end. Dispersed pitting (P). Image size 20 × 20 µm; deflection image. (C) 1.5 cm from the root end. Pitting in the junction area of two scales (P). Image size 10 × 10 µm; deflection image. (D) 4.5 cm from the root end. Pitting in linear layout (P). Image size 10 × 10 µm; deflection image. (E) 3.5 cm from the root end. Absence of striations, absence of ghosts, absence of endocuticle remnants. Small sand‐like globules of 92.5–185.1 nm (green frame). Image size 5 × 5 µm; deflection image. (F) 0.5 cm from the root end. Globule conglomerates of 4290 nm size (green frames). Image size 30 × 30 µm; deflection image.

Scratches were the most prominent feature on the hair of LPP patients. They took various forms and shapes—triangles, lines, and commas. The scratches were usually arranged parallel to the long axis of the hair fiber (Figure 4A–C). Another important feature visualized on the surface of LPP patients' hair was small, circular excavations, so called pits (Figure 5A–D). In our study, pitting occurred along the entire length of LPP hair, but was most commonly located 1.5–4.5 cm from the root end. Single pits were also observed in healthy hair; however, pitting on LPP hair was characterized by a high density of larger and smaller holes. Their clusters were located on specific scales: in some hairs, pitting was also observed in the area where two scales joined (at the base of another scale) or in a characteristic linear arrangement. In contrast, pits on healthy hair were dispersed in nature, far fewer, and all were in comparable size.

Globules were observed in larger numbers in the areas of sudden hair fiber destruction in LPP patients. Three distinct types of globules were visualized: (1) globules with flat elevation and dimensions of hundreds of nanometers; (2) smaller globules (sand‐like) with dimensions of tens of nanometers; and (3) large conglomerates with dimensions of thousands of nanometers. Sand‐like globules were more frequently observed in the LPP group, while the typical flat‐elevation globules were often found in the control group (Figure 5E,F).

### 3.3 Morphological changes unique to the hair of LPP patients

#### 3.3.1 AFM visualization of areas of narrowing and cavities identified by light microscopy

**FIGURE 6:**
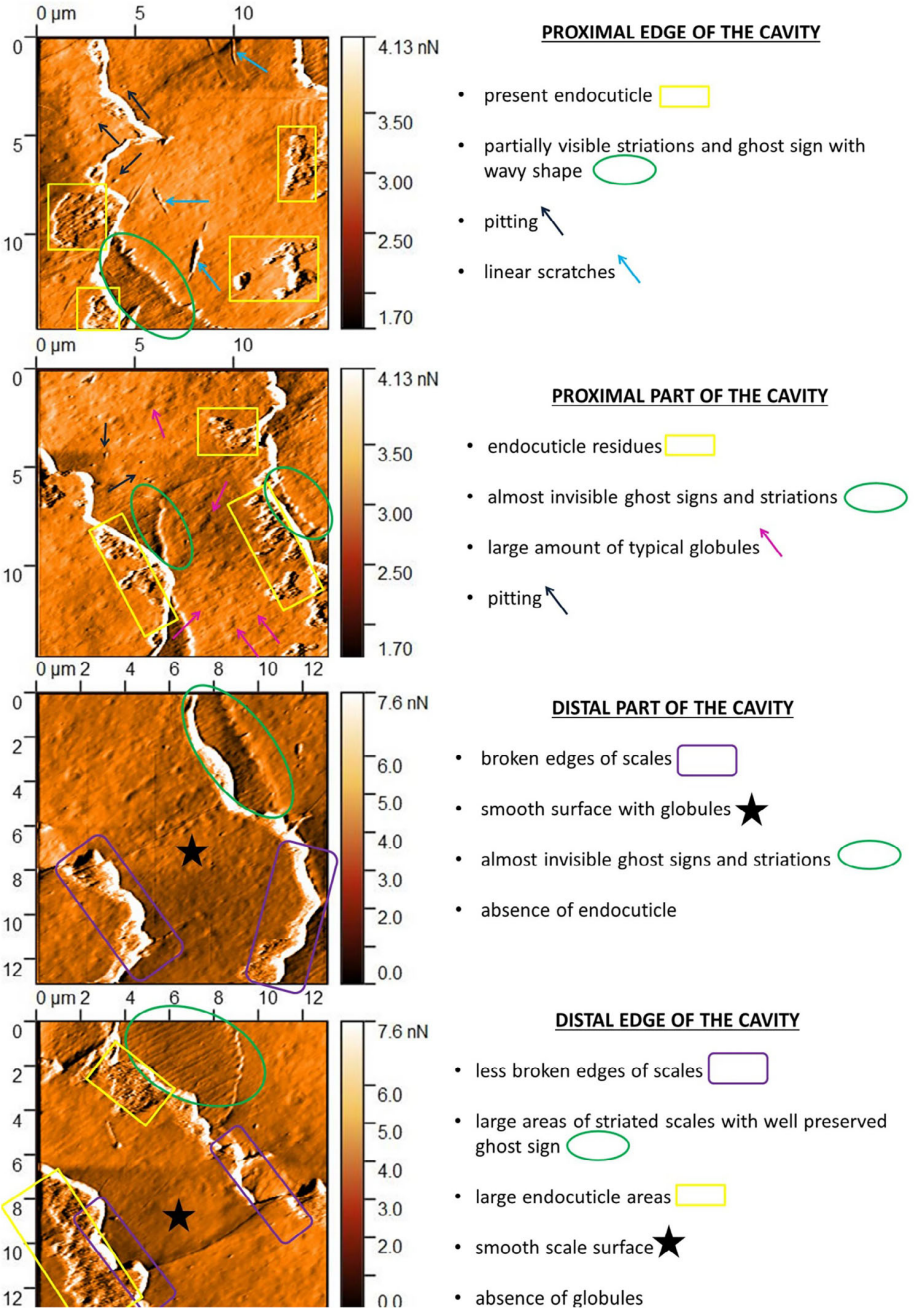
Visualization of surface changes from the area and center of the cavity. Images size from 12.0 × 12.0 µm to 15.0 × 15.0 µm; deflection images.

Using AFM, we found areas of sudden architectural disruption and greater fraying of the edges of the scales compared to further sections of the hair (where the edges of the cells should become increasingly serrated with distance from the root). These areas on lower magnification light microscopy included the narrowing area of the hair fiber and the oval cavities (Figure S1). The oval structures imaged by light microscopy varied in magnitude. We imaged cavities having sizes of 60–100 µm in the long axis and 10 µm in the short axis, as well as with smaller sizes of 10 × 10 µm. With AFM, in areas of LPP hair fiber narrowing and around oval cavities, we detected the acceleration of the delamination process confirmed by ghost signs, increased exfoliation of the cuticle surface, and exposure of the endocuticle. Ghost signs were observed in larger amount in LPP hair compared to healthy hair. The exfoliation was so intense that areas of globules formation were visible, despite prior processing of the preparations. Structural changes in the immediate area of the oval cavities were even more evident. At AFM microscope magnifications, no ghost sign was detected anymore, but only the completely exfoliated top layers of the cuticle and the exposure of the layer beneath the endocuticle (the hair inner layer). The endocuticle itself was visible as a very thin fragment right at the edge of the next scale. Interestingly, the area included either intact scales with smooth contours and superficial striations or much lower areas of the exposed inner layer covered with irregularities (globules) (Figure 6).

#### 3.3.2 Fragments of perifollicular scaling on the surface of the hair

**FIGURE 7:**
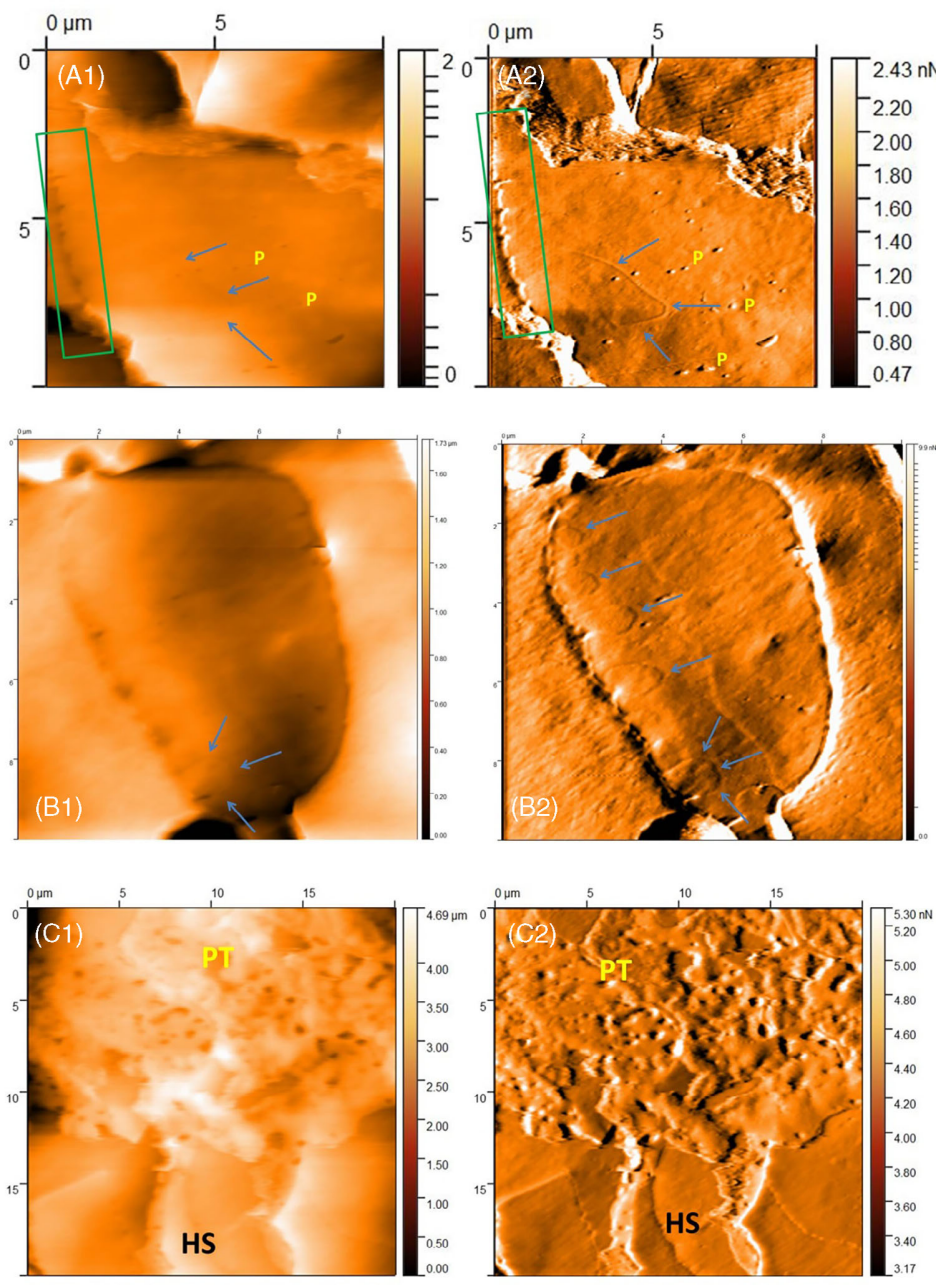
Atomic force microscopy of the hair from patient with lichen planopilaris. (a1‐a2) 2.0 cm from the root end. Linear dent (blue arrows). Wavy edge (green frames). Pitting in linear layout (P). Images size 10 × 10 µm; a1 *Z*‐axis image; a2 deflection image. (b1‐b2) 2.0 cm from the root end. Ellipsoidal structures (blue arrows). Images size 10 × 10 µm; b1 *Z*‐axis image; b2 deflection image. (c1‐c2) 3.5 cm from the root end. Hair scales (HS). Perforated texture on the surface of the hair (PT) (most probably fragments of collar epithelial cells that were destroyed as the hair grew). Image size 20 × 20 µm; c1 *Z*‐axis image; c2 deflection image.

At 0.5 and 1 cm from the root end, undulating soft structures lying on the surface of the hair fiber were visualized. These structures resembled the epithelial cells that were visualized in other studies.^13^ This was also confirmed by the fact that AFM images were taken at the site of the visible collar in the light microscope. At further distances (approximately 3.5 cm), similar structures were observed lying on the surface of the scales, which differed in their perforated texture (Figure 7C). These are fragments of collar epithelial cells that were systematically destroyed as the hair grew. The 3D model showed that these structures protruded above the surface of the hair scale, rather than being embedded in it. This analysis argues more in favor of the fact that the described hair features were remnants of scalp epithelial cells.

#### 3.3.3 Linear indentations and longitudinal cracks

In the study group, single linear indentations arranged in an ellipse shape and in various other unexpected shapes were noted (Figure 7A,B). The lesions described above were less frequently seen on the striated surface and more frequently on the deeper and softer layers of the cuticle.

**FIGURE 8:**
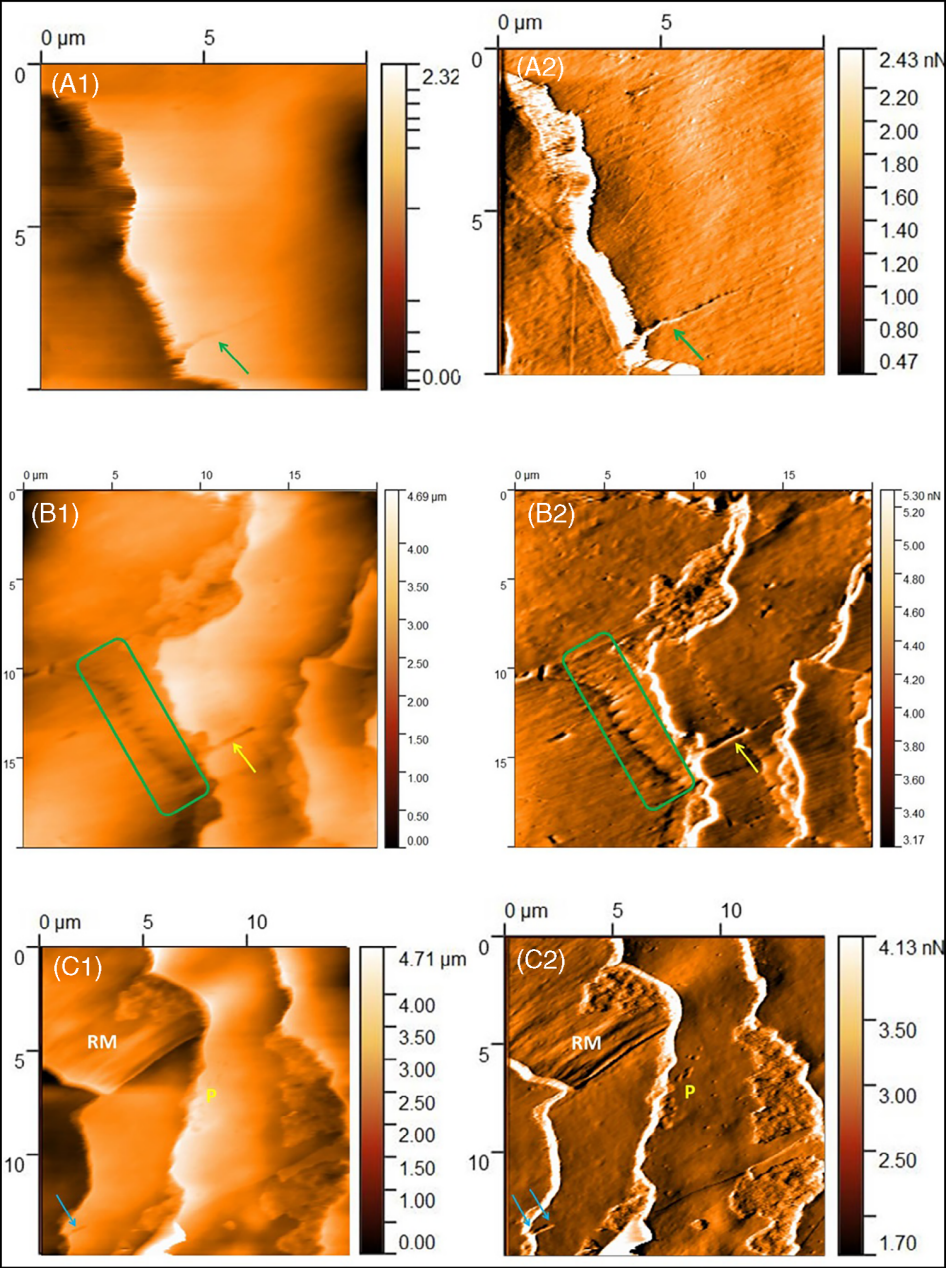
Atomic force microscopy of the hair from patient with lichen planopilaris. (a1‐a2) 1.5 cm from the root end. Linear crack (green arrows). Image size 10 × 10 µm; a1 *Z*‐axis image; a2 deflection image. (b1‐b2) 2.0 cm from the root end. Wavy edge (green frames). Linear crack (yellow arrows). Image size 20 × 20 µm; b1 *Z*‐axis image; b2 deflection image. (c1‐c2) 2.0 cm from the root end. Linear crack (blue arrows). Rod‐like macrofibrillar elements on the side edge of the scale (RM). Smaller and larger holes resembling corrosion pitting (P). Image size 15 × 15 µm; c1 *Z*‐axis image; c2 deflection image.

Cracks were not previously revealed in healthy hair. In diseased hair, it runs parallel to the long axis of the hair fiber. They started from the free end of the scale and fractured inwards. Moreover, cracks were frequently observed in scales with wavy edges (Figure 8A‐C).

## 4 DISCUSSION

LPP and FFA (frontal fibrosing alopecia) constitute major inflammatory lymphocytic subtypes of primary scarring alopecia. As they share the same etiopathogenesis but differ in the clinical presentations, these two variants are “two branches of the same tree.”^14^, ^15^ In both conditions, lymphocytic infiltrate composed mainly of CD8+ T‐cells is implicated in the destruction of hair follicles. LPP and FFA also share other pathogenic mechanisms involving epidermal‐mesenchymal transition and abnormal peroxisome proliferators‐activated receptors (PPAR)‐γ‐mediated signaling.^16^ On the other hand, genetic differences and the influence of environmental factors may be responsible for varying clinical presentation. In classical LPP, the scalp vertex is predominantly involved, while in FFA, the involvement of the frontal and parietal regions leads to the recession of frontal hairline (Figure S2). Both LPP and FFA require early diagnosis and treatment due to largely irreversible damage of the hair follicles and development of scarring alopecia.^15^

In LPP, the perifollicular inflammation and subsequent fibrosis may deform the hair follicle and generate hair shaft abnormalities.^17^, ^18^ Dermoscopy (trichoscopy) constitutes major non‐invasive tool for the preliminary diagnosis of hair and scalp conditions.^19^, ^20^ The main hair abnormality in LPP that may be visualized under dermoscopy is the presence of pili torti, also referred to as “twisted hair.”^17^ Pili torti are defined as irregularly flattened and twisted hair shafts. They may be encountered in genetic diseases such as Netherton syndrome, as well as in acquired conditions, including LPP, discoid lupus erythematosus, folliculitis decalvans, alopecia areata, and primary cutaneous lymphomas.^17^, ^19^, ^21^ However, it should be realized that the role of dermoscopy in assessing the ultrastructural abnormalities of hair shafts is limited. In addition, dermoscopy does not enable the evaluation of the impact of hair shaft changes on its mechanical properties.

In our previous study, we characterized the appearance of healthy hair and defined features indicative of the natural process of hair delamination and destruction as it grows.^12^ The study was performed on the control group presented in this publication (Table 1). The previous results and the created pattern of hair appearance during natural delamination provided a reference for the evaluation of diseased hair. Four different hair fiber surfaces, ghost marks, and changes in the shape of the scales and their free edges were characterized. Hair fiber surfaces such as scales with striated surface, endocuticle, scales with smooth surface, and cortex were identified. Only scales with a striated surface were present on the initial, undamaged sections of the hair. As the top layer of scales detaches (from the free end of the scale), the deeper layers of scales are exposed: endocuticle followed by the smooth surface of the next layer of scales. These lesions were most intense at 1.5–6.5 cm from the root end. The smooth surface gained an advantage over the striated surface at approximately 4.5 cm from the root end. The next layer—the cortex—can be examined at the end tip of the hair. This surface was visible after complete delamination of the cuticular layer with hair scales. In addition, the gradual peeling of the superficially lying scales exposes a depression on the underlying scales—a ghost sign. This symptom was a marker of the transition between the outer striated surface and the deeper smooth surface. The hair scales just at the root end had smooth and unbroken edges. Gradual detachment of the free edges of the scales caused their edges to become increasingly frayed and broken. By breaking off the edges of the scales, the shape of the scales also changed—from convex through flat to concave.^12^

Increased signs of delamination may suggest increased hair destruction in patients with LPP. This may indicate either a weakened hair structure that forms in the histologically altered hair follicle, or it helps to emphasize the timing of the factor influencing hair destruction, which led to the development of the disease even before the onset of clinical symptoms. Wavy edges may develop during hair formation in the hair follicle and the passage of the hair through the inflamed infundibulum and through contact with the surface of the inner root sheath. The scratches and pitting may have been related to the hair's excessive susceptibility to mechanical trauma caused by the disease or as a symptom of high traumatization of the hair, which may happen during the disease. Another possibility is the theory that infiltration of inflammatory cells in the hair follicle, which disrupted its architecture, might cause scratching of the scales that formed.

In 2009, hair surface changes were described in one of the most common types of alopecia—alopecia areata (AA); this was the first study comparing the morphological changes of the hair surface along its length between patients with alopecia areata and a group of healthy individuals.^10^ AA presents with a lymphocytic inflammatory infiltrate in anagen hair follicles, abnormal hair shaft structure, and hair loss occurring without scarring.^22^ Although the disease affects approximately 2% of the general population, few publications are available on the application of the AFM technique in this disease entity. Lee at el.^10^ compared cuticle surface extracted parameters and morphology between a group of healthy hair and hair obtained from patients with AA (*n* = 12 each). They found that the hair surface of patients with AA was more damaged than the control group. Following morphological changes on the surface of the hair scales were described: crack of scale, longitudinal striation, endocuticular ghost, and debris.^10^ These lesions were classified as hair scale damage, but to our knowledge, longitudinal striation is the normal surface of undamaged mammalian hair scales. Scale cracks and endocuticular ghost were also recognized in our study as features indicative of the process of damage to the hair surface. The “debris” described by the authors may correspond to the globules we have observed. In addition, metric measurements of hair scales were taken in the above study. Parameters such as the top distance and step height of cuticle and the curvature of cuticle edge were created and evaluated. Compared to the control group, top distance and step height of cuticle were lower in the group with AA. Meanwhile, the curvature of cuticle edge was higher in the group with AA. In our study, we also used a parameter such as scale deviation measured as scale step heights to assess the hair of LPP patients.^10^ In 2013, Kim et al.^11^ compared the morphology and mechanical properties of the scales of patients with scalp psoriasis (*n* = 14), seborrheic dermatitis (*n* = 28) with a control group (*n* = 50). Scans were performed at one location on each hair (1 cm from the root end). Therefore, it was not possible to follow the dynamics of hair changes with distance from the root in the above study. The researchers described and assessed features such as the pits, the scale thickness, and the surface roughness. They described the pits as a depression in the hair surface. Pits were divided into micropits (diameter < 0.5 µm^2^ or area < 0.25 µm^2^) and macropits (diameter > 0.5 µm^2^ or area > 0.25 µm^2^). Pits were rarely observed in the control group and in patients with seborrheic dermatitis, while it was a common feature in hair with psoriasis. Macropits were demonstrated in 100% of hair samples from psoriasis patients, whereas in the seborrheic dermatitis patients and control group, many of the cavities were micropits. In our LPP group, most of the cavities were macropits (92%). In addition, the researchers showed differences in hair scale thickness between patients with dermatological diseases and hair of healthy subjects.^11^ A similar scale thickness was found in the psoriasis and seborrheic dermatitis patient group. It was approximately four times greater than in the control group. In contrast, the roughness of the hair surface differed significantly between the psoriasis group, where it was greatest, and the control and seborrheic dermatitis groups.^11^ A similar study by Shin et al. in 2012^9^ evaluated the morphology of the hair surface in patients with psoriasis vulgaris. Pitting, roughness, and thickness of hair scales were assessed. They examined hair from healthy subjects and hair from patients with psoriasis vulgaris. In addition, they compared hair from psoriasis patients extracted from active lesions on the scalp and from areas not clinically occupied by the disease process. The researchers found increased scale thickness and a greater number of macropits in psoriasis patients compared to hair from healthy subjects. These features were observed more frequently in both from active psoriasis lesions and hair from non‐lesional skin in the psoriasis patient group. Importantly, by imaging similar nanosized changes in hair growing on the non‐lesional scalp, they showed that psoriasis is generalized.^9^

## 5 CONCLUSION

LPP is difficult to treat, so advances in understanding the molecular mechanisms may contribute to the discovery of new treatments and the possibility of remission in the near future. This is to our knowledge the first comparison study about hair shaft surfaces over the whole lengths between LPP and healthy group using AFM. The study presented here made it possible to assess the dynamics of hair surface changes with distance from the root. This is related to hair growth and different disease onset in different patients. Examining a long section of hair reduced errors due to omission of areas where changes due to disease pathogenesis could potentially be seen.

The results presented in this study attempt to characterize morphological changes at the nanoscale, which may be helpful for early diagnosis of hair diseases in the future. The preliminary results provided give direction for further research into scalp and hair diseases. Our observations indicate that the same structures are present in healthy and diseased hair. The hair does not have a smooth, uniform surface, but a characteristic pattern. The hair of people with LPP and healthy hair have noticeable differences in their surface area. In diseased hair, regions with an increase in structures indicative of damage can be observed, which may suggest the onset of disease. In addition, we observed several structures in LPP absent in healthy hair. The etiology of the structures shown remains to be further discussed. The main limitations of this study were the focus on morphological assessment of the hair surface only and the small sample size. Mechanical properties of the hair surface were not assessed. The lack of software to automatically measure structures on the hair limits the acquisition of numerical data, since the size of the scales, as well as their deviation, is an individual feature. They vary even in a single person depending on the region of the scalp from which the hair was taken and the phase of hair growth. AFM in contact mode is limited to providing images of the top surface of a single fiber. The main limitation of the method is its duration. Image capture time was 6 min at 256 points/line, 12 min at 512 points/line, and approximately 20 min at 1024 points/line. It took about 20 h to examine a single hair, making it unattainable to perform the test on hundreds of patients. Due to the time‐consuming nature of the method and the need to take multiple measurements along the length of a single hair, a small group of individuals was included in the study.

Of value for the above publication is the fact that similar morphological features have already been observed in previous, though few, publications. The pathogenesis of previously tested diseases is similar to LPP, so previous results confirm part of our observations. Therefore, further research in this direction seems reasonable.

## CONFLICT OF INTEREST STATEMENT

The authors declare no conflicts of interest.

## ETHICS STATEMENT

The study was approved by the Ethics Committee of the Rzeszow University (Decision No 9/10/2020 and 6/11/2020). Patients underwent all examinations after they signed informed consent.

## Supporting information

Supporting Information

## Data Availability

Data are available from the corresponding author upon reasonable request.
