# Supplementary material for: Evaluation of hair surface structure and morphology of patients with lichen planopilaris (LPP) by atomic force microscopy (AFM)
Source: Skin Res Technol. 2024 Sep 1;30(9):e70030. doi: 10.1111/srt.70030 (PMC11366445; doi:10.1111/srt.70030)

**Supplementary Figure 1.**

Photography of lichen planopilaris hair under the light microscope: visible oval cavity (yellow circle) and narrowing (blue arrow) of the hair fiber (AFM tip – black asterisk and scanning hair fiber – white asterisk).

**
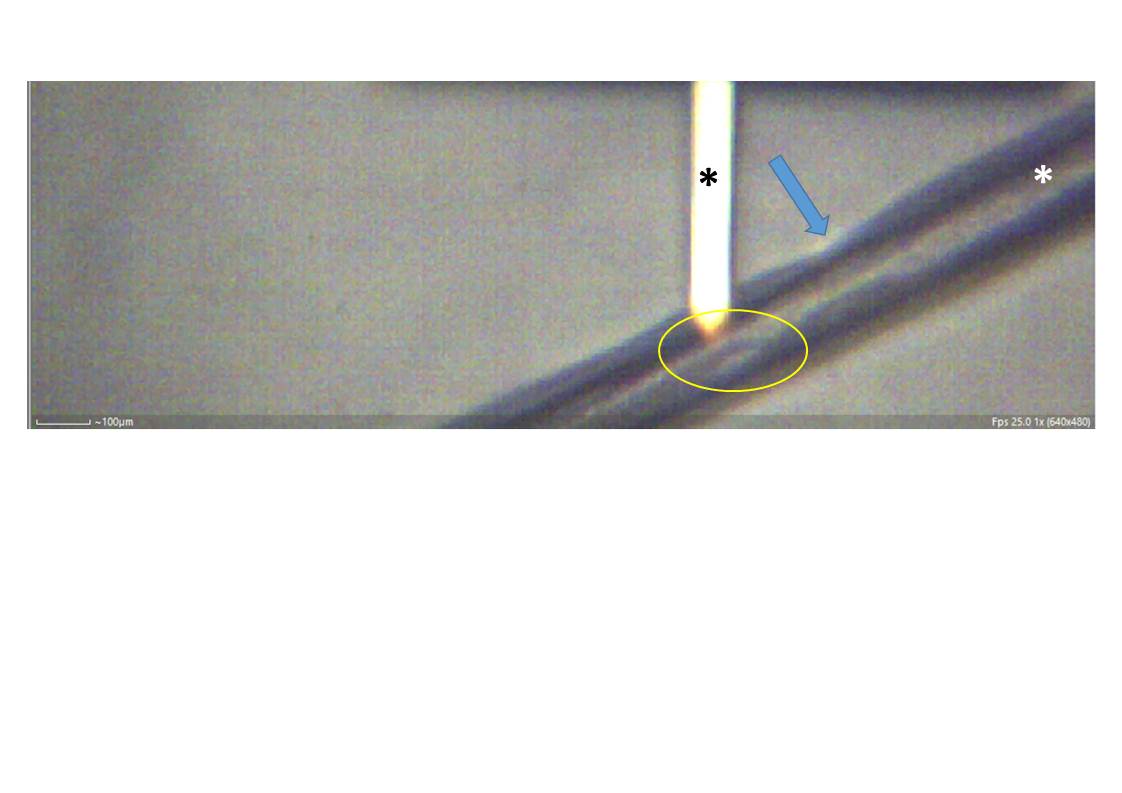
**

**Supplementary Fig. 2.**

Clinical and dermoscopic presentation of lichen planopilaris (LPP).

(a) Erythema and perifollicular scaling (*blue arrows*) on the vertex of the scalp in classical variant of LPP; (b) Frontal fibrosing alopecia (FFA) with marked recession of the frontal hairline (*red asterisk*) and pronounced perifollicular erythema and scaling (*blue arrows*); (c) Corresponding dermoscopic presentation of LPP – extensive perifollicular scaling (*yellow arrows*) on an erythematous background; (d) Corresponding dermoscopic presentation of FFA – perifollicular erythema and scales (*yellow arrows*), single hair shafts in follicle units with absence of vellus hair, structureless areas with absence of follicle ostia (*red asterisk*) confirming the cicatricial nature of alopecia.


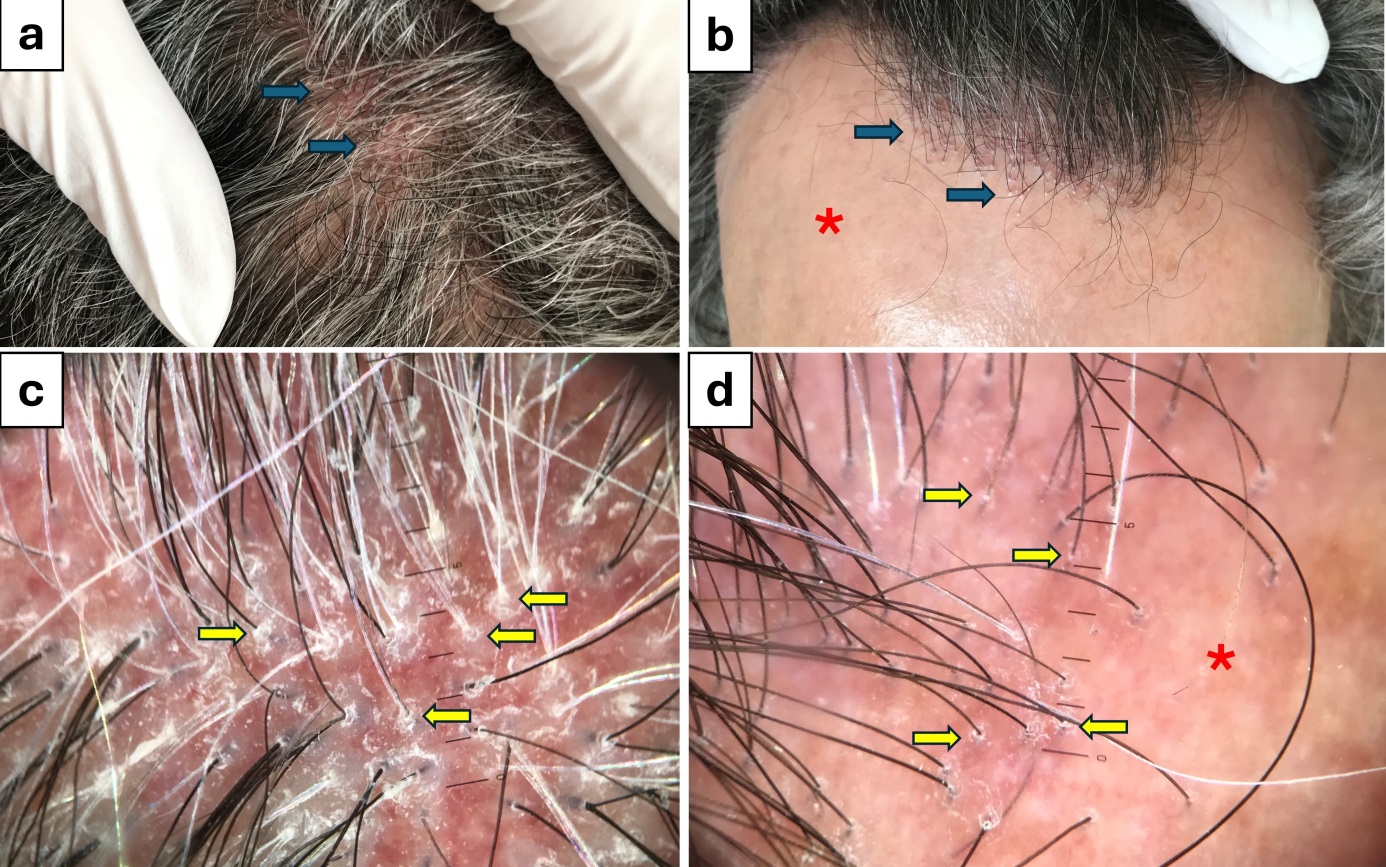

Supplement: Supplementary file 1 — Supporting Information [file SRT-30-e70030-s001.docx]
